# Quality Improvement Project: Monitoring of Intellectual Disability Patients on Anti-Psychotic Medications in Outpatients Clinic, Northern Health & Social Care Trust, Northern Ireland

**DOI:** 10.1192/bjo.2022.293

**Published:** 2022-06-20

**Authors:** Adam Flynn, Patrick Ling

**Affiliations:** Northern Health & Social Care Trust, Antrim, United Kingdom

## Abstract

**Aims:**

Working as part of a newly-established Community Intellectual Disability Team since April 2020, we set a goal of achieving a target of monitoring 90% of patients who attend outpatient's clinic and who are on antipsychotic medications. This includes both physical observations and blood results, inline with NICE Guidelines. On initial analysis, we were essentially starting from a baseline of zero as patients were often deemed too difficult in primary care for monitoring and this simply wasn't happening as a result.

**Methods:**

Retrospective Analysis of patients who attended Outpatients Clinic between February and August 2021. n = 242. Duplicates and Nursing Home Patients were deemed as exclusion criteria.

Analysis via Paris and Electronic Care Record as to which patients were on Antipsychotic medication. n = 73

Analysis of data regarding physical checks and blood records from September 2020–2021 to capture data in line with NICE guidelines.

Liaising with clinical staff to establish any reasons for exclusions, such as a lack of consent. Follow-up of same.

2x PDSA cycles established. One to capture results, and a second involving acquiring new ECG machine and establishing baseline testing, training and analysis of patients.

**Results:**

91% of patients met target criteria of having antipsychotic bloods monitored. Aim 90%.

97% of patients met target of having physical observations monitored. Aim 90%.

Starting from a baseline of zero, we began to capture ECG monitoring of patients from October 2021 and are currently achieving 42% of patients monitored between October 2021 and January 2022 and aim to achieve over 90% by September 2022.

**Conclusion:**

Working as part of a highly-motivated new community team, we have shown that it is clearly possible to achieve a high level of monitoring of patients with mild to profound intellectual disability who are on antipsychotic medications, in line with NICE guidelines.

This has established a new baseline that is a clear and valid evidenced improvement compared to previous standards.

Future monitoring and PDSA cycles will continue to crystalize this data and establish a high standard of care in the community for this patient cohort improving living standards and avoiding and delaying onset of physical health concerns secondary to the cardio-metabolic effects of antipsychotic medications.

